# Dengue virus antibodies enhance Zika virus infection

**DOI:** 10.1038/cti.2016.72

**Published:** 2016-12-16

**Authors:** Lauren M Paul, Eric R Carlin, Meagan M Jenkins, Amanda L Tan, Carolyn M Barcellona, Cindo O Nicholson, Scott F Michael, Sharon Isern

**Affiliations:** 1 Department of Biological Sciences, College of Arts and Sciences, Florida Gulf Coast University, Fort Myers, FL, USA

## Abstract

For decades, human infections with Zika virus (ZIKV), a mosquito-transmitted flavivirus, were sporadic, associated with mild disease, and went underreported since symptoms were similar to other acute febrile diseases. Recent reports of severe disease associated with ZIKV have greatly heightened awareness. It is anticipated that ZIKV will continue to spread in the Americas and globally where competent *Aedes* mosquito vectors are found. Dengue virus (DENV), the most common mosquito-transmitted human flavivirus, is both well-established and the source of outbreaks in areas of recent ZIKV introduction. DENV and ZIKV are closely related, resulting in substantial antigenic overlap. Through antibody-dependent enhancement (ADE), anti-DENV antibodies can enhance the infectivity of DENV for certain classes of immune cells, causing increased viral production that correlates with severe disease outcomes. Similarly, ZIKV has been shown to undergo ADE in response to antibodies generated by other flaviviruses. We tested the neutralizing and enhancing potential of well-characterized broadly neutralizing human anti-DENV monoclonal antibodies (HMAbs) and human DENV immune sera against ZIKV using neutralization and ADE assays. We show that anti-DENV HMAbs, cross-react, do not neutralize, and greatly enhance ZIKV infection *in vitro*. DENV immune sera had varying degrees of neutralization against ZIKV and similarly enhanced ZIKV infection. Our results suggest that pre-existing DENV immunity may enhance ZIKV infection *in vivo* and may lead to increased disease severity. Understanding the interplay between ZIKV and DENV will be critical in informing public health responses and will be particularly valuable for ZIKV and DENV vaccine design and implementation strategies.

Zika virus (ZIKV), a mosquito-transmitted flavivirus, was first isolated in a sentinel rhesus monkey and *Aedes africanus* mosquitoes in the Zika Forest near Entebbe, Uganda in 1947 during routine arbovirus surveillance by the Virus Research Institute in Entebbe.^1^ Simpson described the first well-documented case of ZIKV disease and virus isolation in humans.^2^ In 1968, ZIKV was isolated from three non-hospitalized children in Ibadan, Nigeria, indicating that ZIKV was not restricted to East Africa.^3^ A 1953 and 1954 serological survey in South East Asia that included individuals from Malaysia near Kuala Lumpur, Thailand and North Vietnam found ZIKV protective sera in individuals residing in these regions ranging from 75% positive in Malayans, 8% in Thailand and 2% in North Vietnam.^4^ An early 1980s serologic study of human volunteers in Lombok, Indonesia reported that 13% had neutralizing antibodies to ZIKV.^5^ These studies illustrated that ZIKV had spread beyond Africa and at some point became endemic in Asia.^6^

For decades, human ZIKV infections were sporadic, spread in geographic location, remained associated with mild disease and perhaps went underreported since its symptoms were similar to other acute febrile diseases endemic in the same regions.^7^ As is the case with other flaviviruses, it is known that ZIKV antibodies cross-react with other flavivirus antigens including dengue virus (DENV) as was illustrated in the Yap State, Micronesia ZIKV outbreak in 2007. Initial serologic testing by immunoglobulin M (IgM) capture ELISA with DENV antigen was positive which led physicians to initially conclude that the causative agent for the outbreak was DENV, though the epidemic was characterized by a rash, conjunctivitis and arthralgia symptoms clinically distinct from DENV.^8^ Subsequent testing using a ZIKV-specific reverse transcriptase–PCR(RT–PCR) assay revealed that ZIKV was the causative agent.^9^ No further transmission was reported in the Pacific until 2013 when French Polynesia reported an explosive ZIKV outbreak with 11% of the population seeking medical care.^10^ Perinatal ZIKV transmission was also reported in French Polynesia.^11^ In addition, 3% of blood bank samples tested positive for ZIKV by RT–PCR even though the donors were asymptomatic when they donated, underscoring the potential risk of ZIKV transmission through blood transfusions.^12^ ZIKV transmission and spread maintained a solid foothold in the Pacific^13^ and continued its spread in 2014 with confirmed outbreaks in French Polynesia, New Caledonia, Easter Island and the Cook Islands.^14, 15, 16, 17^

The first report of local transmission of ZIKV in the Americas occurred in 2015 in the city of Natal in Northern Brazil.^18^ Natal patients reported intense pain resembling chikungunya virus (CHIKV) infection but with a shorter clinical course, in addition to maculopapular rash. No deaths or complications were reported at the time, though given the naive immunological status of the Brazilian population, ZIKV expansion was predicted. By mid-January 2016, ZIKV transmission had occurred in 20 countries or territories in the Americas as reported to the Pan American Health Organization.^19^ The primary mode of ZIKV transmission appeared to be through mosquito vectors, although cases of perinatal and sexual transmission were also reported.^11, 20^ Given its recent history of rapid spread in immune naive populations, it is anticipated that ZIKV will continue to spread for the foreseeable future in the Americas and globally in regions where competent *Aedes* mosquito vectors are present. Kindhauser *et al.*^21^ can serve as a comprehensive account of the world-wide temporal and geographic distribution of ZIKV from 1947 to present day.

Until relatively recently, due to its mild clinical outcome, ZIKV disease had not been a critical public health problem. As a result, compared with other related viruses, it remained understudied. However, recent reports of severe ZIKV disease including Guillain-Barré syndrome in French Polynesia^13, 22^ and associations between ZIKV and microcephaly and other severe fetal abnormalities in Brazil^23, 24, 25, 26, 27^ have greatly heightened awareness of ZIKV. Retrospectively, the incidence of Guillain-Barré syndrome during the 2014 ZIKV French Polynesia outbreak and the incidence of microcephaly in Brazil in 2015 were both 20 times higher than in previous years. The cause of these severe ZIKV disease outcomes remains an open question. Recent ZIKV outbreaks in the Pacific and the Americas have been explosive and associated with severe disease, yet earlier expansions in Africa and Asia were gradual, continuous and associated with mild clinical outcomes. Much of the difference may lie in the age of exposure. In an endemic scenario where many adults have pre-existing ZIKV immunity, new cases would then primarily occur in children. Introduction of ZIKV into immune naive populations where all ages are susceptible to infection, including women of child-bearing age, is the new scenario for ZIKV expansion in the Americas. However, we are still left without an understanding of why certain individuals develop severe disease such as Guillain-Barré syndrome, and why some expectant mothers transmit ZIKV to their developing offspring *in utero*, resulting in fetal infection and developmental abnormalities, whereas others do not. A possible explanation could be the impact of pre-existing immunity to co-circulating flaviviruses.

Globally, DENV is the most common mosquito-transmitted human flavivirus^28^ and is both well-established and the source of new outbreaks in many areas of recent ZIKV introduction.^14, 15^ DENV and ZIKV are very closely related resulting in substantial antigenic overlap. The four serotypes of DENV (DENV-1, DENV-2, DENV-3 and DENV-4) have an antigenic relationship that impacts disease severity. Infection with one serotype typically results in a life-long neutralizing antibody response to that serotype, but yields cross-reactive, non-neutralizing antibodies against the other serotypes. These cross-reactive, non-neutralizing antibodies are responsible for antibody-dependent enhancement (ADE), a phenomenon where DENV particles are bound (opsonized) by these antibodies, which facilitates the infection of antibody Fc receptor (FcR) bearing cells. The presence of enhancing antibodies correlates with increased DENV viremia and disease severity.^29, 30, 31^ Similarly, ZIKV has also been shown to undergo ADE in response to sub-neutralizing concentrations of homologous anti-serum, and in response to heterologous anti-serum from several different flaviviruses.^32^ In addition, anti-ZIKV sera has been shown to enhance infectivity of related viruses.^33^ In one study, immune mouse ascites against various flaviviruses including ZIKV, West Nile virus (WNV), Yellow Fever-17D (YF17D), Wesselsbron virus, Potiskum, Dakar Bat, and Uganda S were tested for ZIKV ADE in P388D_1_, a mouse macrophage Fc receptor cell line.^32^ All heterologous immune mouse ascites, as well as homologous immune ascites, enhanced ZIKV in culture. Of note, the fold-enhancement was greater for ZIKV compared with peak enhancement of other flaviviruses tested against their heterologous immune ascites. Given the incidence of co-circulating flaviviruses, the study authors alluded to the importance of testing human sera for ADE potential of circulating flaviviruses. In a subsequent study, human cord blood and sera of newborns and adults collected in Ibadan, Nigeria, was tested for ADE of DENV-2, YF17D and WNV in P388D_1_, but the ADE potential of ZIKV was not tested.^34^ Curiously, the 2013–14 French Polynesia ZIKV outbreak demonstrated that all the patients with Guillain-Barré syndrome had pre-existing DENV immunity.^22^

In this study, we investigated the role that pre-existing DENV immunity plays during ZIKV infection. Here we report that human anti-DENV serum and well-characterized human anti-DENV monoclonal antibodies (HMAbs) cause substantial ZIKV ADE in a human Fc receptor bearing cell line. Our results suggest that pre-existing antibodies from a prior DENV infection may enhance ZIKV infection *in vivo* and may increase disease severity.

## Results

### Cross-recognition of ZIKV E protein by human anti-DENV antibodies

It is well known that infection with closely related flaviviruses often results in a cross-reactive serum antibody response. The primary neutralizing epitopes targeted by human antibodies during a flavivirus infection are found in the envelope glycoprotein (E protein).^35, 36, 37, 38, 39, 40, 41, 42^ The role of the E protein is to facilitate virus entry by binding and mediating the fusion of the virus membrane and cellular membrane in target cells. The E protein of ZIKV and the four serotypes of DENV have a high degree of genetic similarity and the amino acid sequence of fusion loop region of these viruses is identical. In a previous study, we characterized broadly neutralizing anti-DENV human monoclonal antibodies (HMAbs) derived from patients that had recovered from DENV infection.^42^ These HMAbs recognized the E protein with high affinity, neutralized the four DENV serotypes, and mediated ADE *in vitro* at subneutralizing concentrations. Their neutralization activities correlated with a strong inhibition of intracellular fusion, rather than virus-cell binding. In addition, we mapped epitopes of these HMAbs to the highly conserved fusion loop region of the E protein.

**Figure 1:**
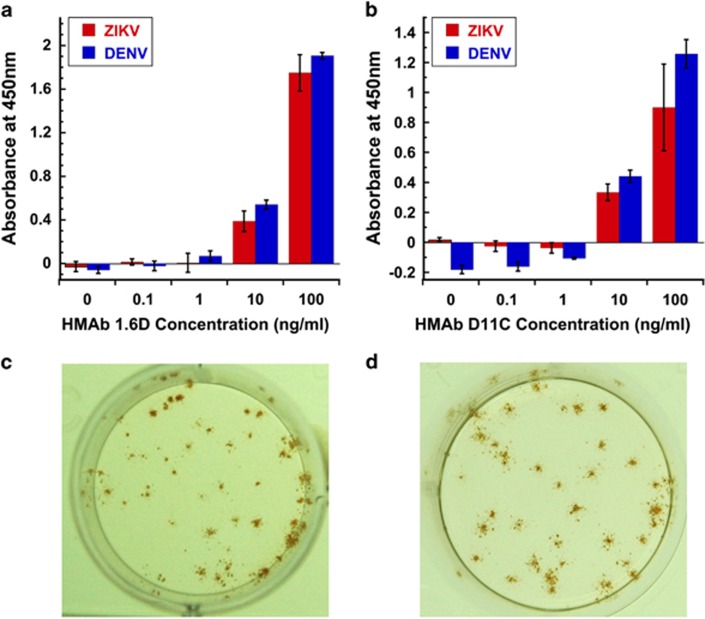
ELISA and immunostaining cross-reactivity of anti-DENV HMAbs against ZIKV. Anti-DENV HMAbs 1.6D and D11C that recognize the DENV E protein fusion loop cross-react with ZIKV MR766 strain E surface glycoprotein as shown by ELISA (**a**, 1.6D; **b**, D11C) and recognize ZIKV MR766 infected cells in an immunostained focus-forming assay (**c**, 1.6D; **d**, D11C). DENV E is serotype 2, strain New Guinea-C. Absorbance from no antigen control reactions was used to normalize the results. ELISA data shown are representative of two independent assays each done in triplicate. 10 and 100 ng ml^−1^ concentrations were statistically significantly higher than no antigen controls by analysis of variance with Dunnett's *post hoc*, *P*<0.05.

Given the high degree of similarity between the DENV E protein and the ZIKV E protein, we thus tested the ability of two of these well-characterized anti-DENV HMAbs, 1.6D and D11C, to recognize the glycosylated ZIKV E surface protein using a ConA capture assay.^42^ In this assay, the glycoprotein-binding lectin, ConA, is used to capture ZIKV MR766 E glycoprotein, which is then recognized by anti-DENV HMAbs that recognize the DENV E protein fusion loop. The HMAb is then detected with an anti-human IgG HRP-conjugated secondary antibody and an HRP colorimetric substrate. Our results show that anti-DENV HMAbs, 1.6D and D11C, strongly recognize the ZIKV E surface glycoprotein (Figures 1a and b). In addition, we tested the ability of these HMAbs to recognize ZIKV-infected cells in an immunostained focus forming assay (Figures 1c and d). This result confirms that anti-DENV E fusion loop HMAbs cross-react with ZIKV.

### *In vitro* ZIKV neutralization activity of broadly neutralizing anti-DENV HMAbs

**Figure 2:**
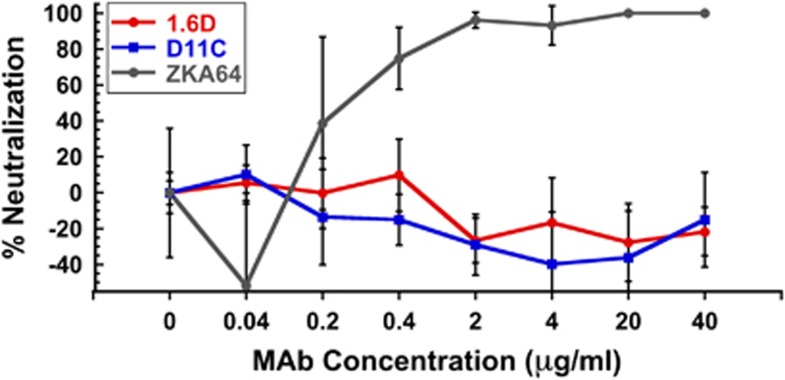
Infectivity neutralizing activity of anti-DENV HMAbs against ZIKV MR766. Broadly neutralizing anti-DENV HMAbs 1.6D and D11C do not inhibit ZIKV MR766 infection in LLC-MK2 cells at the concentrations tested. However, a positive control neutralizing anti-ZIKV antibody (ZKA64) showed clear concentration-dependent inhibition. The results shown are the average±the s.d. of six replicates. None of the concentrations of HMAbs 1.6D or D11C tested showed statistically significant neutralizing activity compared to no antibody controls by analysis of variance with Dunnett's *post hoc*, *P*>0.05.

Since anti-DENV HMAbs 1.6D and D11C were crossreactive against ZIKV, we tested whether they could neutralize ZIKV infectivity using an immunostained focus-forming unit reduction neutralization assay in rhesus macaque LLC-MK2 kidney epithelial cells.^42^ Fusion loop HMAbs D11C and 1.6D are broadly neutralizing against all four DENV serotypes and represent a very common class of broadly neutralizing HMAbs, perhaps the dominant broadly neutralizing class of antibodies against DENV.^42^ However, neither 1.6D nor D11C inhibited ZIKV infectivity *in vitro* at the concentrations tested (up to 40 μg ml^−1^), while a commercially available anti-ZIKV neutralizing antibody showed clear concentration-dependent inhibition (Figure 2). Broadly neutralizing anti-DENV HMAbs that target the E protein fusion loop bind to ZIKV antigens, but do not neutralize infectivity.

### *In vitro* ZIKV enhancement activity of broadly neutralizing anti-DENV HMAbs

**Figure 3:**
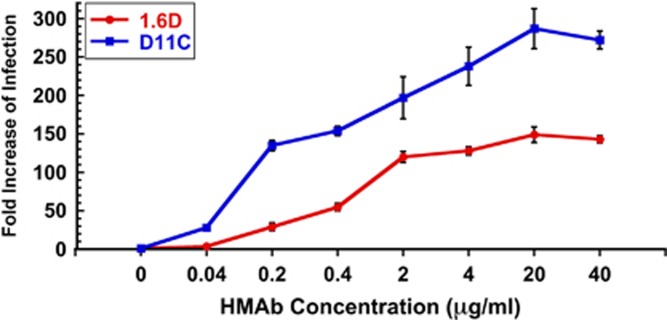
Enhancing activity of anti-DENV HMAbs against ZIKV by qRT–PCR. Broadly neutralizing anti-DENV HMAbs 1.6D and D11C show strong ZIKV MR766 infection enhancing activity. Independent assays were repeated twice in triplicate. All concentrations above 0.2 μgml^−1^ were statistically higher than no antibody controls by analysis of variance with Dunnett's *post hoc*, *P*<0.05.

DENV ADE of Fc receptor (FcR)-bearing cells, which include macrophages, monocytes, and dendrocytes, correlates with increased viremia and severe disease outcomes.^43^ Antibodies that recognize DENV surface proteins, but do not neutralize infectivity, can direct viral binding and infection of certain FcR cells that are not normally infected. Since anti-DENV HMAbs 1.6D and D11C cross-reacted with ZIKV proteins, but did not neutralize ZIKV infection, we tested whether they could mediate ZIKV ADE *in vitro*. In Figure 3, we show that ZIKV infection of FcR-bearing K562 cells can be strongly enhanced by anti-DENV HMAbs 1.6D (~140-fold) and D11C (~275-fold). Virus genome yields measured by quantitative RT–PCR (qRT–PCR) in the absence of HMAbs were in the order of 10^3^ genome equivalents compared to 10^5^ genome equivalents for peak enhancement in the presence of HMAbs.

### *In vitro* ZIKV neutralization activity of human anti-DENV serum

Given the cross-reactive and strongly enhancing potential of anti-DENV HMAbs 1.6D and D11C, we investigated whether immune sera from DENV recovered patients contained other types of antibodies that could neutralize ZIKV infection. For this study, we wanted to investigate what might be considered the ‘worst case scenario' with regards to pre-existing immunity to DENV. We selected sera from individuals with secondary DENV infection that had been collected in countries where multiple serotypes of DENV have been known to circulate. This scenario would serve to model the immune status of many individuals in regions where ZIKV is rapidly spreading.

**Figure 4:**
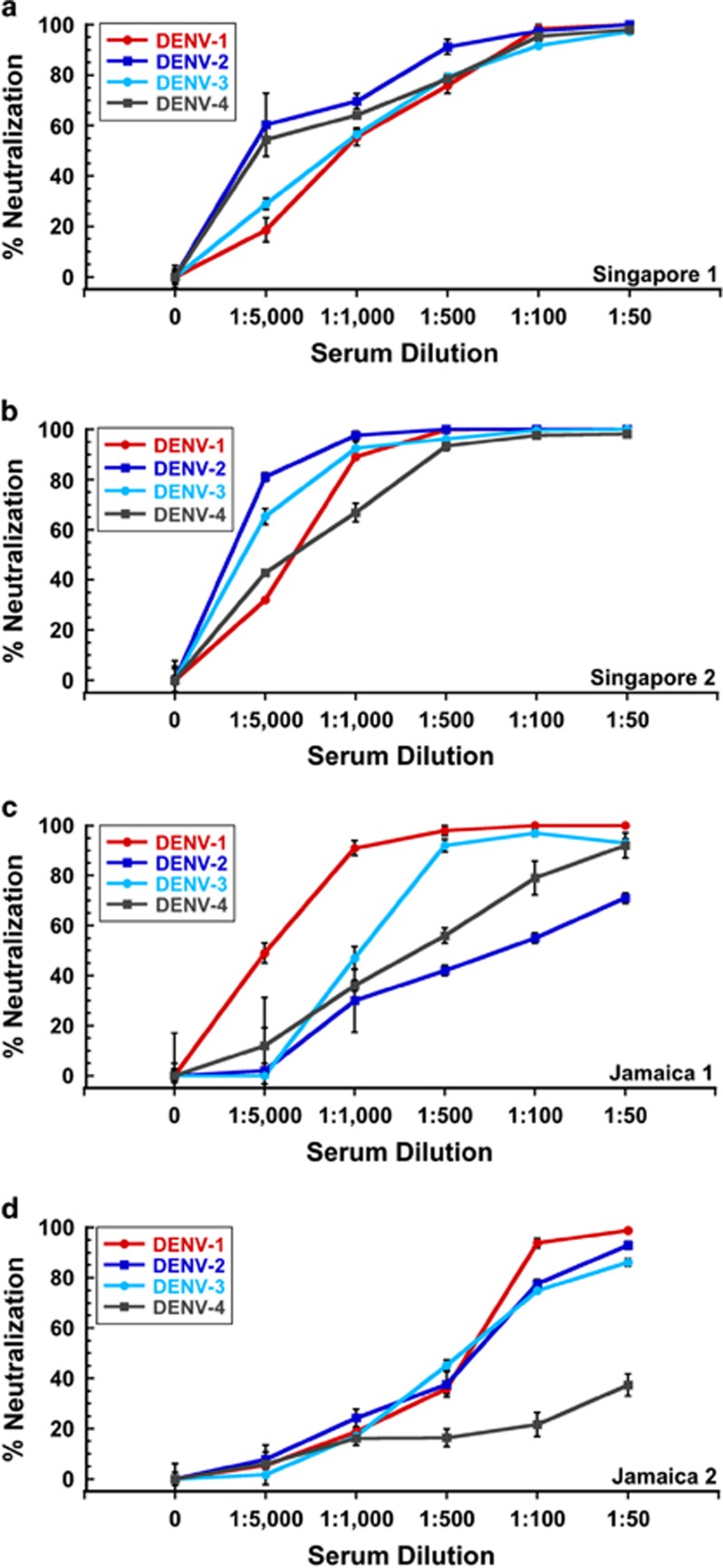
Infectivity neutralizing activity of anti-DENV human sera against DENV. All anti-DENV human sera showed broad neutralizing activity against multiple DENV serotypes 1–4.^42^ (**a**) Singapore 1, (**b**) Singapore 2, (**c**) Jamaica 1 and (**d**) Jamaica 2. All concentrations above 1:1000 dilution showed statistically significant inhibition of infection compared to no serum controls by analysis of variance with Dunnett's *post hoc*, *P*<0.05.

We tested two human anti-DENV sera from Singapore and two from Jamaica, in addition to serum from a DENV-negative donor from Canada. The Singapore patient sera were collected in 2008 during which time ZIKV was endemic in Southeast Asia and after its expansion in the Yap State in Micronesia in the Pacific in 2007. The Canada donor serum was collected in 2003 and the Jamaica sera were collected in 2008 prior to documented introduction of ZIKV in the Americas. Additionally, the Jamaica and Canada subjects had no travel history to ZIKV endemic countries. We purposely selected Singapore 1 and Jamaica 1 sera for these studies since subject Singapore 1 was the source of HMAb D11C and subject Jamaica 1 was the source of HMAb 1.6D.^42^ We wanted to determine whether the antibody repertoire of the same individuals contained DENV antibodies that could also neutralize ZIKV infection. Singapore 2 and Jamaica 2 sera were selected based on their broadly neutralizing activity against DENV. As shown in Figure 4, the Singapore (1 and 2) and Jamaica (1 and 2) sera showed broadly neutralizing activity against all four serotypes of DENV,^42^ indicating that they were likely from subjects with secondary DENV infections.

**Figure 5:**
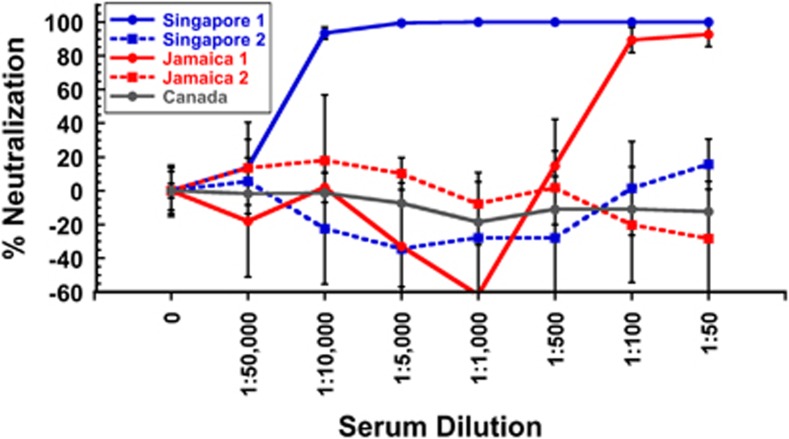
Infectivity neutralizing activity of anti-DENV human sera against ZIKV. Human anti-DENV sera from Singapore and Jamaica showed both non-neutralizing and neutralizing activity against ZIKV MR766. Singapore 1 serum strongly neutralized ZIKV MR766, suggesting prior ZIKV infection, while Singapore 2 serum had no neutralizing activity. Jamaica 1 serum neutralized ZIKV MR766 at high serum concentrations, while Jamaica 2 serum showed no neutralizing activity at the dilutions tested. Control serum from Canada showed no ZIKV neutralizing activity. The results shown are the average±the s.d. of six replicates. All dilutions of Singapore 1 serum as well as 1:100 and 1:50 dilutions of Jamaica 1 serum showed statistically significant inhibition of infectivity by analysis of variance with Dunnett's *post hoc*, *P*<0.05. No other sera showed statistically significant infectivity neutralizing activity.

The results of the ZIKV neutralization assays with human anti-DENV sera are shown in Figure 5. We found that Singapore 1 serum strongly neutralized ZIKV, even at high dilutions (1:10 000 dilution), while Singapore 2 had no ZIKV neutralizing activity. Jamaica 1 serum neutralized ZIKV at the highest serum concentrations tested (1:100, 1:50), while Jamaica 2 serum did not. We suspect that the strongly ZIKV neutralizing Singapore 1 serum may be the result of a prior undiagnosed ZIKV infection, as ZIKV has been present in Southeast Asia for decades.^4, 5, 21^ However, the less potent neutralizing activity from Jamaica 1 serum is very likely due to cross-neutralization from prior DENV infection, or infections, as ZIKV was unknown in the Americas at the time the serum was collected. Serum from Canada with no exposure to DENV or ZIKV was used as a negative control and had no ZIKV neutralizing activity.^44^

### *In vitro* ZIKV enhancement activity of human anti-DENV serum

**Figure 6:**
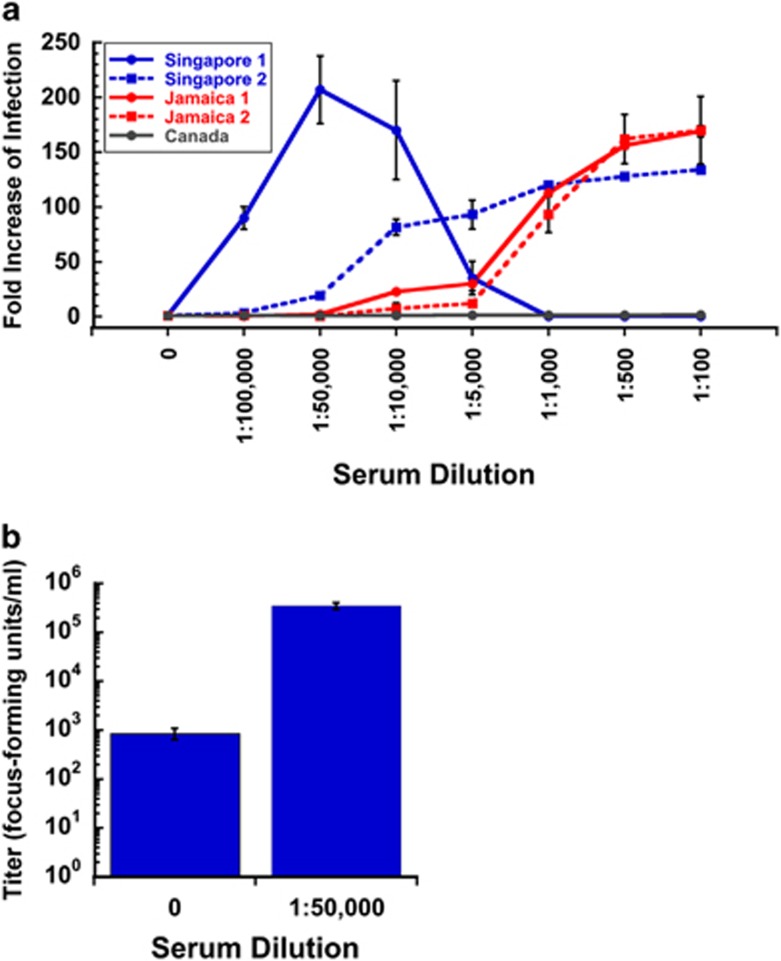
Enhancing activity of anti-DENV human sera against ZIKV by qRT–PCR and infectious particle assay. (**a**) The effect of anti-DENV human sera on enhancement of ZIKV MR766 infection was determined by qRT–PCR in the human FcRII bearing cell line K562. All human anti-DENV sera tested showed strong infection enhancing activity of ZIKV MR766. At high serum concentrations, Singapore 1 serum blocked enhancement due to its strong neutralizing activity. Independent assays were repeated twice in triplicate. Compared with no serum controls, Singapore 1 serum showed statistically significant levels of enhancement at 1:100 000–1:10 000 dilutions, Singapore 2 serum at 1:50 000 and lower dilutions, Jamaica 1 serum at 1:10 000 and lower dilutions, and Jamaica 2 serum at 1:1000 and lower dilutions by analysis of variance with Dunnett's *post hoc*, *P*<0.05. Canadian serum showed no statistically significant effect at any dilution. (**b**) The effect of anti-DENV human sera on the production of infectious progeny viral particles was determined in the human FcRII bearing cell line K562 infected with ZIKV MR766 in the presence (1:50 000 dilution) or absence (0=no serum control) of Singapore 1 serum. The presence of enhancing serum resulted in a large and statistically significant increase in the amount of infectious progeny virus released (*T*-test, *P*=0.00016).

We then tested whether human DENV immune sera could mediate ADE *in vitro*. We show that ZIKV infection of FcRII bearing K562 cells can be strongly enhanced (up to 200-fold) by all human anti-DENV sera tested (Figure 6a). In comparison, the control serum from Canada showed no enhancement. The highly neutralizing Singapore 1 serum showed strong ZIKV enhancement at intermediate dilutions (1:100 000 to 1:10 000) that diminished at lower dilutions (1:5 000 to 1:100), indicating that highly neutralizing antibodies can overcome ZIKV infection enhancement at sufficiently high concentrations.

To understand the relationship between replication of viral RNA in infected cells, which is measured by qRT–PCR, and production of viral progeny, we conducted assays to measure the amount of infectious virus produced from antibody-enhanced infections versus non-enhanced infections. In Figure 6b, we show that FcRII cells infected with ZIKV strain 766 in the absence of serum produced only approximately 800 focus-forming units per ml, while cells infected with ZIKV MR766 in the presence of a 1:50 000 dilution of Singapore 1 serum produced approximately 300 000 focus-forming units per ml, an increase of over two orders of magnitude.

**Figure 7:**
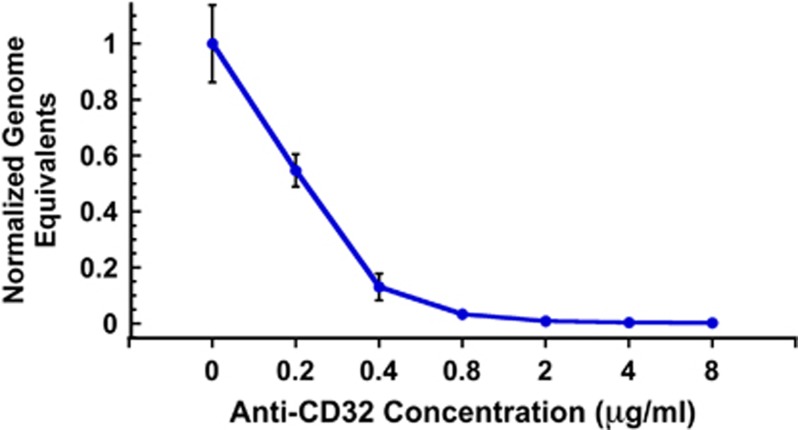
Anti-FcRII antibody blocks ZIKV enhancement activity of anti-DENV serum. K562 cells were pre-incubated with increasing concentrations of mouse anti-FcRII (CD32) MAb prior to infection with ZIKV MR766 that had been pre-incubated with a highly enhancing dilution (1:50 000) of Singapore 1 serum. The results indicate that the ZIKV enhancement effect can be effectively blocked in a dose-responsive manner with an anti-FcRII MAb. This effect was statistically significant at all concentrations by analysis of variance with Dunnett's *post hoc*, *P*<0.05.

To confirm that the mechanism of enhancement involved entry of antibody-bound ZIKV particles through the K562 FcRII pathway, we pre-incubated K562 cells with a mouse anti-FcRII MAb prior to infection with ZIKV that had been pre-incubated with a highly enhancing dilution (1:50 000) of the ZIKV-neutralizing Singapore 1 serum. Our results demonstrate that the ZIKV enhancement effect can be effectively blocked in a dose-responsive manner with an anti-FcRII MAb (Figure 7).

**Figure 8:**
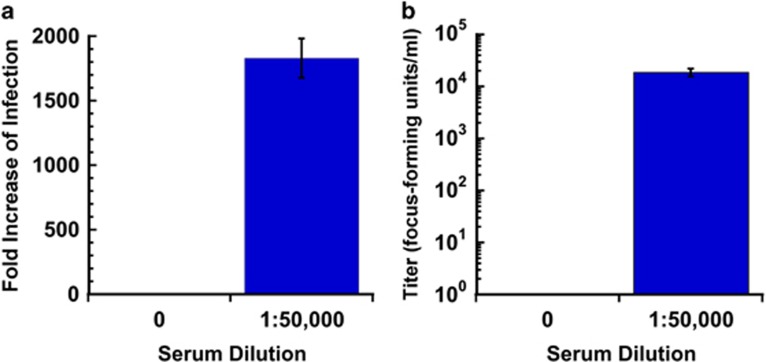
Enhancing activity of anti-DENV human sera against ZIKV PRVABC59 by infectious particle and qRT–PCR assays. The effect of anti-DENV human sera on the production of (**a**) replication of viral RNA and on (**b**) infectious progeny viral particles was determined in the human FcRII bearing cell line K562 infected with ZIKV PRVABC59 in the presence (1:50 000 dilution) or absence (0=no serum control) of Singapore 1 serum. The presence of enhancing serum resulted in a large and statistically significant increase in the amount of viral RNA (*T*-test, *P*=0.000015) as well as infectious progeny virus released (*T*-test, *P*=0.00077).

To confirm that the ADE results we have shown were not an artifact restricted to the use of ZIKV strain MR766, we also tested the enhancement sensitivity of a recent 2015 ZIKV isolate PRVABC59 from Puerto Rico. In Figure 8 we show by both qRT–PCR (Figure 8a) and production of infectious progeny (Figure 8b) that infection by ZIKV strain PRVABV59 was greatly enhanced. In the presence of a 1:50 000 dilution of Singapore 1 serum, a >1800-fold increase genome copies was observed in the cell pellet by qRT–PCR and a 4 orders of magnitude increase in focus-forming units per ml (0 focus-forming units per ml in the absence of serum and 1.88 × 10^4^ focus-forming units per ml in the presence of serum) was detected in the cell culture supernatant.

## Discussion

The present scenario of ZIKV introduction and spread in the Pacific and the Americas is complicated by pre-existing immunity to DENV. A recent serological survey of women giving birth in 2009–2010 in central Brazil documented that 53% of the new mothers were IgG positive for DENV.^45^ ZIKV enhancement has been previously described to occur in the presence of cross-reactive sera raised against other flaviviruses. Here we demonstrate that broadly neutralizing anti-DENV E protein fusion loop HMAbs cross-react with ZIKV, do not neutralize ZIKV, and greatly enhance the replication of viral RNA and the production of viral progeny of ZIKV in cultured FcRII-positive cells. Although the 10 amino acid E protein fusion loop region itself is identical between DENV and ZIKV, the binding epitope for these HMAbs is likely to be much larger and include important interactions with other variable portions of the E proteins that impact neutralization activity. We noted previously that these two HMAbs show little or no neutralizing activity against YFV or WNV.^42^

In this study, we also investigated the role of sera from secondary DENV infections that might be considered as the worst-case scenario in DENV endemic regions. Our results show that human sera from secondary DENV infections can show varying degrees of activity, from neutralizing to non-neutralizing, and similarly enhance ZIKV infection. We have confirmed that the *in vitro* mechanism of ZIKV enhancement occurs through an FcRII-dependent process in human K562 cells in a manner very similar to DENV. We also show that the serum-mediated enhancement effect occurs for both an African and a recent American strain of ZIKV. If ZIKV ADE is fundamentally similar to DENV ADE, it is highly likely that pre-existing anti-DENV antibodies will increase ZIKV viremia in humans and lead to more severe disease *in vivo*. This correlation will need to be confirmed clinically.

These results have implications for our understanding of ZIKV spread and persistence. In areas where DENV is endemic, ZIKV may transmit more readily and persist more strongly than expected from epidemiological transmission models of ZIKV alone, as has been observed in the recent ZIKV expansion in the Pacific and the Americas. How this plays out as ZIKV continues to spread in the Americas and other parts of the world where competent *Aedes* mosquito vectors are present, remains to be seen. One hopeful possibility is that ZIKV spread may be slower in areas where DENV immunity is low.

These results also have consequences for DENV and ZIKV vaccine design and use. We identified two serum samples that showed neutralizing activity against both DENV and ZIKV. The activity of highly neutralizing Singapore 1 serum is most likely explained by prior, undiagnosed ZIKV infection.^7^ Whereas the Jamaica 1 serum neutralizing activity is likely not due to prior ZIKV infection, but may be a combined response against multiple DENV infections. In any case, this raises the possibility of inducing dual ZIKV and DENV immunity, perhaps with a single vaccine. Although the broadly neutralizing, anti-DENV HMAbs we tested did not neutralize ZIKV, there may be other human antibodies that may recognize and neutralize both ZIKV and DENV.^46^ However, DENV vaccines that induce a broadly reactive antibody response against viral surface envelope proteins with a large non-neutralizing antibody component may result in a cross-reactive, enhancing response against ZIKV, especially as the vaccine response wanes over time. Additionally, we know little about the reciprocal response of anti-ZIKV antibodies and their capacity to enhance DENV infections, although it would seem plausible that anti-ZIKV antibodies might similarly enhance DENV. A clear understanding of the interplay between ZIKV and DENV infections will be critical to ZIKV planning and response efforts in regions where ZIKV and DENV co-circulate, and particularly valuable for vaccine design and implementation strategies for both ZIKV and DENV. Note added in proof: After we posted the results of our study to *bioRxiv*,^47^ two groups independently reported similar results showing that serum and monoclonal antibodies from Thai cohorts with previous DENV infections caused enhancement of ZIKV infection.^48, 49^ Albeit, the pooled sera used in those studies was collected in locations where both ZIKV and DENV may likely have co-circulated during the time of collection.

## Methods

### Ethics statement

The collection of human blood samples was reviewed and approved by the institutional review board of Florida Gulf Coast University (protocols 2007–08 and 2007–12) and the research ethics committee of the Centre Hospitalier de l'Université de Montréal. Informed written consent was obtained from all subjects.

### Human sera and monoclonal antibodies

Jamaica 1, and Singapore 1 sera have been previously described, from subject 8C and subject DA003, respectively.^42^ Subject Jamaica 1 (8C) was infected with DENV in Jamaica in 2007 and had blood drawn in 2008, ~3 months post recovery. The subject had fever for 12 days, headache, retro-orbital pain, and blood in sputum. Subject Jamaica 2 (10E) was infected with DENV in Jamaica in 2007 with severe symptoms and had blood drawn in 2008, 3 months after recovery. Subject Singapore 1 (DA003) was hospitalized in Singapore in 2008 for complications due to DENV infection and had blood drawn ~4 weeks post-recovery. No hemoconcentration or bleeding was present. Subject Singapore 2 (PHC) was infected with DENV and hospitalized in Singapore in 2008 and had blood drawn ~4 weeks after recovery. A healthy subject from Montreal, Canada provided control serum that was collected in 2003 prior to vaccination with yellow fever 17D vaccine. Travel history confirmed that the subject had not travelled to regions outside North America and had no previous exposure to DENV or ZIKV. Sera were heat inactivated for 30 min at 56 °C prior to use. Anti-DENV HMAbs 1.6D and D11C isolated from subject Jamaica 1 and Singapore 1, respectively, were kindly provided by JS Schieffelin from Tulane University and have been well-characterized and described previously.^42^ Anti-ZIKV antibody ZKA64 (Absolute Antibody, Oxford, UK, catalog # Ab00779-2.0) was used as a positive control for neutralization.

### Viruses and cell culture

The 1947 Ugandan isolate, ZIKV MR766, and DENV-1 strain HI-1, DENV-2 strain New Guinea-2, DENV-3 strain H-78 and DENV-4 strain H-42, were kindly provided by RB Tesh at the University of Texas at Galveston through the World Reference Center for Emerging Viruses and Arboviruses. The 2015 Puerto Rican ZIKV isolate, PRVABC59 was kindly provided by the Centers for Disease Control and Prevention, Arbovirus Branch. ZIKV stocks were propagated by single passage in African green monkey (*Cercopithecus aethiops*) kidney epithelial cells, Vero (ATCC CCL-81, American Type Culture Collection, Manassas, VA), cultured in Eagle's Minimum Essential Medium supplemented with 10% (v/v) fetal bovine serum (FBS), 2 mM Glutamax, 100 U ml^−1^ penicillin G, 100 μg ml^−1^ streptomycin and 0.25 μg ml^−1^ amphotericin B at 37 °C with 5% (v/v) CO_2_. Rhesus macaque (*Macaca mulatta*) kidney epithelial cells, LLC-MK2 (ATCC CCL-7) used to propagate DENV and titer and perform focus-forming unit reduction neutralization assays, were cultured in Dulbecco's Modified Eagle Medium (DMEM) supplemented with 10% (v/v) FBS, 2 mM Glutamax, 100 U ml^−1^ penicillin G, 100 μg ml^−1^ streptomycin and 0.25 μg ml^−1^ amphotericin B at 37 °C with 5% (v/v) CO_2_. Human FcRII-expressing K-562 cells from a monocyte-granulocyte lineage with lymphoblast morphology (ATCC CCL-243) were cultured in RPMI-1640 (Hyclone, Logan, UT) supplemented with 10% (v/v) FBS, 2 mM Glutamax, 100 U ml^−1^ penicillin G, 100 μg ml^−1^ streptomycin and 0.25 μg ml^−1^ amphotericin B at 37 °C with 5% (v/v) CO_2_. All reagents were purchased from ThermoFisher, Waltham, MA, USA, unless otherwise noted.

### ELISA

ELISA were performed as follows. Corning brand high-bind 96-well plates (ThermoFisher, Waltham, MA, USA) were coated with 100 μl Concanavalin A (ConA) (Vector Laboratories, Burlingame, CA, USA) at 25 μg ml^−1^ in 0.01 M *4*-(2-hydroxyethyl)-1-piperazineethanesulfonic acid (HEPES) (Sigma, Saint Louis, MO, USA) and incubated for 1 h at room temperature. Wells were washed with phosphate buffered saline (PBS) with 0.1% (v/v) Tween 20 (Sigma) and incubated for 1 h at room temperature with 100 μl of filtered ZIKV or DENV-2 culture supernatant inactivated with 0.1% (v/v) Triton-X100 (Sigma). After a wash step with PBS containing 0.1% (v/v) Tween 20, wells were blocked with 200 μl PBS containing 0.5% (v/v) Tween 20 and 5% (w/v) non-fat dry milk for 30 min. Primary HMAbs D11C and 1.6D in PBS containing 0.5% (v/v) Tween 20 were incubated for 30 min at room temperature. After a wash step, 100 μl of a peroxidase-conjugated affinity purified anti-human IgG (Pierce, Rockford, IL) diluted to 1 μg ml^−1^ in PBS-0.5% (v/v) Tween 20 was incubated at room temperature for 30 min to detect the primary antibody. After a final wash step, color was developed with tetramethylbenzidineperoxide (ProMega, Madison, WI, USA) as the substrate for peroxidase. The reaction was stopped after 3 min by adding 100 μl 1 M phosphoric acid (Sigma), and the absorbance was read at 450 nm. Negative controls included media without virus antigen, ConA only, and ConA without primary or secondary antibodies. Absorbance from no antigen control reactions was used to normalize the results.

### Focus-forming assay

Focus-forming assays were performed essentially as previously described.^42^ LLC-MK2 target cells were seeded at a density of ~500 000 cells in each well of a 12-well plate 24–48 h prior to infection. For titer assays, 10-fold serial dilutions of virus were prepared. For neutralization assays, ~100 focus-forming units of virus were incubated with dilutions of heat-inactivated serum or purified HMAbs in serum-free DMEM for 1 h at 37 °C. Mixtures were allowed to infect confluent target cell monolayers for 1 h at 37 °C, with rocking every 15 min, after which the inoculum was aspirated and cells were overlaid with fresh Minimum Essential Medium (MEM) supplemented with 10% (v/v) FBS, 2 mM Glutamax, 100 U ml^−1^ penicillin G, 100 μg ml^−1^ streptomycin and 0.25 μg ml^−1^ amphotericin B containing 1.2% (w/v) microcrystalline cellulose Avicel (FMC BioPolymer, Philadelphia, PA). The infected cells were then incubated at 37 °C with 5% (v/v) CO_2_ for 48 h (DENV-4), 60 h (ZIKV), or 72 h (DENV-1, -2, and -3). Cells were fixed in Formalde-Fresh Solution (ThermoFisher), either overnight at 4 °C or for 1 h at room temperature and permeabilized with 70% (v/v) ethanol for 30 min. Foci were detected using 1 μgml^−1^ primary HMAbs 1.6D or D11C incubated for 8 h at room temperature, followed by secondary horseradish peroxidase-conjugated goat anti-human IgG (H+L) (Pierce, Rockford, IL, USA) incubated for 8 h at room temperature. Foci were visualized by the addition of 3,3-diaminobenzidine tetrahydrochloride (Sigma-Aldrich, St Louis, MO).

### Antibody-dependent enhancement assays

Antibody-dependent enhancement assays were performed as previously described.^42, 50^ Briefly, 250 focus-forming units of ZIKV MR766 or PRVABC59 were mixed with human sera or HMAbs and RPMI medium in a 200 μl volume and incubated for 1 h at 37 °C. Mixtures were added to 80 000 K562 cells in 300 μl of complete RPMI medium and incubated for 3 days at 37 °C, 5% (v/v) CO_2_. Control experiments were performed by pre-incubating cells for 1 h at 37 °C with a mouse anti-human FcRII MAb (anti-CD32) (Biolegend, San Diego, CA). Cells were collected by centrifugation and total RNA was isolated using an RNeasy Mini-kit (Qiagen, Valencia, CA) following the manufacturer's protocol. qRT–PCR was performed on isolated RNA using ZIKV-specific forward (5′-CTGCTGGCTGGGACACCCGC-3′) and reverse (5′-CGGCCAACGCCAGAGTTCTGTGC-3′ for MR766) (5′-GGCCAATGCCAAGGCCC-3′ for PRVABC59) primers to amplify a 98 bp product for MR766 or a 99 bp product from PRVABC59 in the ZIKV NS5 region. A Roche LightCycler 480 II was used to run qRT–PCR using a LightCycler RNA Master SYBR Green I kit (Roche, Indianapolis, IN). Amplification conditions were as follows: reverse transcription at 61 °C for 40 min, denaturation at 95 °C for 30 s, followed by 45 cycles of denaturing at 95 °C for 5 s, annealing at 47 °C (for MR766) or 55 °C (for PRVABC59) for 10 s, and extension at 72 °C for 15 s. For antibody-dependent enhancement assays measuring infectious particle production, infected K562 supernatant media was used immediately to make 10-fold serial dilutions and plated to measure viral titer as described for the focus-forming assay above.
